# Generating viewsheds based on the Digital Surface Model (DSM) and point cloud

**DOI:** 10.1371/journal.pone.0312146

**Published:** 2024-12-31

**Authors:** Jerzy Orlof, Paweł Ozimek, Piotr Łabędź, Adrian Widłak, Agnieszka Ozimek

**Affiliations:** 1 Faculty of Computer Science and Telecommunications, Cracow University of Technology, Cracow, Poland; 2 Faculty of Architecture, Cracow University of Technology, Cracow, Poland; Atlantic Technological University, IRELAND

## Abstract

Visual analysis has applications in diverse fields, including urban planning and environmental management. This study explores viewshed generation using two distinct datasets: Digital Surface Model (DSM) and LiDAR (Light Detection and Ranging) point cloud data. We assess the differences in viewsheds derived from these sources, evaluating their respective strengths and weaknesses. The DSM accurately captures terrain features and elevation changes, offering a comprehensive view of the land surface. Conversely, LiDAR point cloud data delivers detailed three-dimensional information, enabling precise mapping of terrain features and object detection. Our comparative analysis based on six selected locations with varied topographical arrangements considers factors such as visual acuity and computational efficiency. Additionally, we discuss the application of DSM and LiDAR point cloud data in view analysis, emphasizing their value in line-of-sight assessments and field operations. The results indicate greater precision of viewsheds created based on LiDAR point clouds. The analysis reveals that the greater precision in comparing differences between DSM and point LiDAR data ranges from 1.42% to 5.94%, while the results subtraction falls between 1.05% and 3.89% for the conditions analyzed, indicating a high degree of accuracy in the method. However, this process demands significant computational resources. It is best applied in limited areas, particularly in urban environments where such data is crucial for supporting research decisions.

## Introduction

In many scientific studies, authors focus on developing data processing algorithms, with the primary goal of improving the process itself while neglecting the accuracy of the data being used. This oversight can lead to reduced reliability of the results, which was a key aspect of research in the past [1, 2]. In extreme cases, such an approach can result in the omission of significant landscape elements, affecting the quality and completeness of the analysis. Lower data accuracy not only limits processing capabilities but also reduces the potential benefits of advanced analytical methods. For instance, in the case of DSM data, ignoring important attributes, such as point classifications, prevents their full utilization. Such neglect can lead to the loss of critical information, ultimately impacting the quality and accuracy of spatial analysis results. Some researchers have focused on modeling vegetation for visibility analysis but have not examined how the precision of these models affects the accuracy of visibility results [3].

An essential component of any spatial development project is its aesthetics. It manifests itself in the landscape that we perceive through views. The goal of landscape architecture is to create spaces that preserve vistas of both man-made structures and natural features. Protecting the landscape is crucial, particularly in areas where tourism plays a significant role in the local economy. These locations draw visitors because of the well-known sights from the media and postcards. The preservation of beautiful vistas is as vital as the preservation of historical sites, monuments, or the environment. Its main objective is to keep the view from being obstructed by newly constructed human structures and overgrown by carelessly or incorrectly planted vegetation. Via computer vision modeling, the effects of planned changes and land development on the visibility of important landscapes can be precisely determined. While designing the spatial development, existing states are modeled to capture the relationships between their components and to indicate or confirm the places of landscape perception. Design variants are also modeled to demonstrate the impact of transformations on the environment and to select the optimal scenario. A key element of this modeling is viewshed. It is generated for the points, lines, axes, and viewing planes that constitute the places of landscape perception [4]. These are active exposure viewsheds. They disjunctively depict what is and is not visible from a specific place. In passive exposure of elements in which visibility is examined; viewsheds show the points from where a particular space element is visible. Visibility maps are assembled using viewsheds created for collections of points dispersed across surface and linear objects. They have a spectrum of values based on how big the visible part is [11]. To construct visibility maps, from one to even several million viewsheds are needed, depending on the extent of space elements and the assumed sampling accuracy. Therefore, the speed of generating viewsheds and the accuracy of results are crucial. Point clouds are the source of data—accurate, but extremely large. Working with these data consumes a lot of time and processing power. Since DSM and DTM are produced by generalizing point clouds, they are less precise but smaller in size and don’t require as much time or power. The comparison of viewsheds produced using these various data sets aims to show which analyses require the usage of point clouds and for which DSM/DTM is sufficient.

Land planning is essential for effective land management. Data processing is a major component of contemporary urban space management techniques, such as Digital Elevation Models (DEM) [5] or, in general, Digital Surface Models (DSM) [6]. One crucial component of surface topography and a prerequisite for many applications is the DEM. The U.S. Geological Survey USGS defines it as a “digital representation of the land’s elevation at regularly spaced intervals in both the *x* and *y* directions, with *z*-values referenced to a common vertical datum” [7].

While the USGS provides a focused and standardized definition, the broader literature reflects the diversity of formats, sources, and applications for DEMs across different disciplines (DEMs’ applications in terrain analysis, hydrology, and urban planning). Wilson and Gallant [8] describe DEMs as “a tool for understanding and analyzing surface processes by providing a digital model of the terrain.”. Moore et al. (1991) [9] describe a DEM as “a representation of the Earth’s surface derived from remotely sensed data or ground surveys, used for terrain analysis.

Digital Elevation Models (DEMs) and Digital Surface Models (DSMs) are valuable tools in urban planning and management, offering a range of practical applications. DEMs help planners understand the natural topography of an area. Both DEMs and DSMs are used to conduct viewshed analysis, which is essential in assessing the visual impact of new developments, such as high-rise buildings, on existing landscapes and neighborhoods. This analysis helps in ensuring that new structures do not obstruct important views or degrade the aesthetic value of an area. The detailed surface information provided by DSMs, including buildings and vegetation, helps in designing transportation networks and utility lines that avoid existing structures and optimize routes for efficiency and safety. Moreover this type of data helps studying sunlight exposure and shadow patterns in urban environments, planning of infrastructure such as roads, bridges, and utilities. Additionally, DEMs are commonly used in hydrological modeling to predict water flow and identify areas at risk of flooding. Urban planners use this information to design drainage systems, flood defenses, and to inform building codes that mitigate flood risks in vulnerable areas [10].

However, in addition to the elevation of the terrain, vegetation and man-made structures must be considered in spatial assessments, particularly those carried out in metropolitan settings. Thus, in contrast to DEM, which simulates the elevation of the land surface without vegetation or buildings, DSM is used in this kind of analysis [11].

LiDAR is a scanning technique that gathers data about objects using electromagnetic radiation beams. According to several researchers [12, 13] it has mostly been linked to large-scale applications like the creation of wide-area DSMs. The most crucial source of LiDAR data for building large-scale DSMs is ALS (Airborne Laser Scanning) [14–16]. TLS (Terrestrial Laser Scanning) is a static, ground-based scanning technique with very high accuracy (up to a few millimeters), ideal for detailed mapping of small areas. UAV LiDAR uses drones, offering mobility and flexibility, though limited by equipment weight and battery life. It’s especially useful in hard-to-reach areas. Handheld LiDAR, or handheld scanners, are highly accurate over small areas but less efficient over larger surfaces due to the time required. Our study focuses on ALS data, which allows for effective large-area mapping.

Kurczynski [14] presents methods for processing large point clouds from ALS to create DSMs with high-quality parameters. He focuses on identifying the key technical parameters of ALS scanning, such as point cloud density, georeferencing accuracy, weather conditions, and the method of dividing the area into LiDAR blocks. The proposed procedure enables data acquisition from large areas, ensuring the best DSM representation in relation to the actual terrain.

J. N. Negishi [15] demonstrated the practical application of ALS in creating DSMs for the analysis of surface connectivity of floodplain water bodies. Although the LiDAR data used had a spatial resolution of only 1 point per 4 m² and vertical and horizontal accuracies of 1 m and 0.25 m, respectively, the study confirmed the high precision of LiDAR in characterizing floodplain terrain and in calculating the frequency of connections between water bodies and the main water channel based on DSM data.

Gao et al. [16] particularly focus on evaluating which interpolation method provides the best results for accurately representing terrain surfaces based on ALS data. The main conclusions are that DEM interpolation accuracy depends on surface topography, the interpolation method, and point cloud density. Moreover, point cloud density has a minimal impact on DEM accuracy in gentle terrains, while uneven terrains exhibit a sharp decline in accuracy with higher data extraction rates. The study highlights that the Kriging method performs best in complex terrains.

The employed methodology facilitates the acquisition of comprehensive land cover information [17]. Landform mapping is based on four principles: the morphologic, the genetic, the chronologic, and the dynamic. Minár, referring to these principles [17], emphasizes the importance of terrain segmentation and geomorphological mapping in the context of varying topographic relief. Classification algorithms that categorise land cover types based on the height and intensity of LiDAR returns also play a crucial role.

Minár, presents research that demonstrates different types of land discontinuities that can be used to refine the segmentation of LiDAR data into meaningful land cover classes. The techniques used in segmenting DEMs from LiDAR-derived data, based on these theoretical principles, enable the creation of more detailed and accurate land cover information by linking specific land forms to their corresponding land cover types.

Furthermore, the mapping of relief in the terrain and penetration of foliage is made possible using laser equipment [18]. Numerous variables influence the accuracy of DSM creation using LiDAR-derived data. Among these are: land cover, sampling density, and surface complexity [19, 20]. Kraus [21] proposed an empirical relationship that states that the DSM precision is proportional to the square root of the point density.

Other models and theories either build upon or offer alternative views regarding the relationship between DSM precision and point density. Höhle et al. [22] discuss an error propagation model in DEM/DSM creation, which, beyond point density, considers factors such as sensor characteristics, terrain roughness, and data processing methods. These models suggest that DSM precision is influenced by a combination of factors, not solely by point density, which contrasts with the simplified view presented by Kraus. Some papers also explore how machine learning can improve the precision of DSMs [23]. This approach has moved away from simple empirical formulas and instead relies on data-driven models that can capture non-linear relationships between point density and DSM precision.

The development of ALS systems leads to increased efficiency, lower data acquisition costs, and the creation of products such as DEMs and DSMs. We are seeing a rapid development of ALS airborne laser scanning technology, of which numerical height models are a typical product. A numerical terrain model generated on the basis of ALS data was until recently a very expensive product; currently, in European conditions, its cost for large projects is around 200 €/km2. In terms of accuracy, ALS has virtually no competition and in this respect is displacing traditional methods, including the development of DSMs from large-scale aerial photographs [14]. To illustrate the practical use of this type of data, it is worth to highlight two cases that demonstrate its increased efficiency. Research in Belgium compared different methods of tree measurement, including ALS, static terrestrial laser scanning (STLS), and manual measurements [24]. The study found that ALS provided comprehensive data on large forest areas quickly and at a much lower cost compared to traditional ground methods. ALS significantly reduced the time required for data acquisition while offering a reliable estimation of forest parameters like tree diameter at breast height. Another case study of flood inundation to the River Meuse in the Netherlands demonstrated increased efficiency achieving an 85.5% accuracy in flood prediction for high-resolution DEMs [25].

As a result, models become more accurate and there is interest in using this method on a wider scale, as evidenced by national studies [14, 26–29]. The European Union’s current legal framework mandates that member states create planning documents for flood risk management [30] or construct a spatial information infrastructure (INSPIRE Directive [31]). Reaching this objective requires the utilization of high-resolution DSM data.

LiDAR data is highly accurate, often requiring significant computing power and disk space for processing and storage [32]. However, generating DEM data with excessive accuracy can limit its applicability, particularly for institutions with smaller budgets. Therefore, interpolating directly acquired LiDAR data is essential when converting them into numerical terrain models. The conversion of LiDAR data into DTM Digital Terrain Model—a digital representation of the bare-earth surface, excluding vegetation, buildings, and other surface features is a subject that is frequently covered in the literature. (e.g. [16, 33–39]).

Gao used different methods to interpolate ALS to DEM for complex, three selected terrains with varying degrees of slope [16]. His research highlights that DEM interpolation accuracy depends on surface topography, method, and point cloud density. Kriging interpolation method is best for rough terrains, while NN excels in gentle terrains.

Hone-Jay Chu et al [34]. and Imran Ashraf et al. [38] state that the choice of interpolation method significantly impacts the accuracy of the resulting DTMs. Different methods yield varying results, particularly in complex terrains. The results presented by them was interpolated with Inverse Distance Weighting (IDW), Kriging and Nearest Neighbor (NN). Both stated, that accurate scarp identification relies on both high point density and the appropriate interpolation technique.

Moreover, Imran Ashraf et al. highlight the importance of using LiDAR intensity data effectively to enhance the resolution and accuracy of DTMs, particularly in areas with complex topography [34].

Key insights based on [33, 35–39] indicate that for complex or rugged terrains, the Kriging method is the most suitable, with TIN also being highly effective. For smooth or moderately varied terrains, IDW, Bilinear Interpolation, and Natural Neighbor are recommended. For discrete data or flat terrains, Nearest Neighbor can be applied, although it is less commonly used for DTMs.

Although some researchers attempt to estimate the theoretical precision of DTM [40, 41], experimental work based on case studies is by far the most common approach [32, 42–45]. The theoretical error aligns with the study’s results and can be represented as the sum of errors from the various stages of DTM creation relative to the point cloud. This includes data acquisition methods, propagation, interpolation [46], and filtering methods. Each of these steps introduces its source of error, which collectively impacts the accuracy of the final DTM model. Data interpolation is accomplished through various algorithms, with the most popular being: Inverse Distance Weighted (IDW) [44, 47–49], Kriging [50–52], spline-based interpolators [47–50], Triangular Irregular Network (TIN) interpolation [19, 53] or Nearest Neighbour. The results can differ at other users since all results depend on the user defined filtering parameters. Therefore, with a more suitable decided parameters, the results can be more accurate. Besides, it is hard to exactly advice any interpolation method as the best method for rasterization based on the results of this study. But it is possible to prefer the NN method for analyzing the terrain features in either application approaches. Because it has higher accuracy and correlation over 90% for all decimations. The generated DEMs with NN can be used for some urban based applications that are required terrain analysis such as communications, energy, agriculture, environmental management, mining, forestry, and emergency management. For future studies, these approaches can be applied to get a better conclusion for filtering methods at different decimation rates and of course the suitable interpolation methods [54, 55]. Although comparing DEMs obtained by different interpolation methods is important [54, 56–59], it is likewise crucial to evaluate the quality of the subsequent data generated from the resulting DEMs or DSMs [60, 61].

The research project aims to compare the potential applications of DSM and point clouds acquired by LiDAR technology to create viewsheds—a graphical representation that distinguishes between visible and invisible areas from a specific point in terrain, commonly used in urban planning and environmental management. It helps assess visual impacts, identify scenic locations, and influence property values. Viewsheds can be generated using either Digital Surface Models (DSM) or point cloud data. This process will be explained further in the Material and Methods section.

Even slight differences in the generated viewsheds can be significant, especially in urbanized areas. Comparison of the results allowed us to assess the accuracy and specifics of the area designated as the visibility range from a given location.

The aim of this study is to demonstrate that the commonly accepted use of DEM data does not always lead to a fully accurate analysis reflecting the actual situation. In our research, we focus particularly on the unique issue of data accuracy, highlighting significant differences between DEM data and the real terrain. Through our analysis and comparison of various data sources, we strive to identify the best practices for achieving the most reliable results.

## Materials and methods

### Digital Surface Model

Discrete measurement data from a digital land cover model can be utilized by an interpolation technique to create a surface shape that spans infrastructure, buildings, trees, bridges, and viaducts. DTM, which was created from aerial photos and gathered as part of Poland’s cyclical coverage with a digital orthophotomap, covers the entire country and is required for the LPIS (Land Parcel Identification System), a component of the IACS (Integrated Administration and Control System). The precision of its altitude ranges from RMSE = 0.9 to 1.5 m, which is inadequate for many needs related to terrain study [14]. This led to the establishment of the ISOK (National Land Cover IT System) [62] initiative, which gathers significantly more precise data. The so-called Standard II represents city regions, where the source data density is not lower than 12 points/m^2^ (two independent transverse ALS flights, each with a density of ≥ 6 points/m^2^, height accuracy of *m*_*h*_ ≤ 0.10*m* and registration of four beam reflections). Similar projects in Europe usually use lower densities (0.5-1.0 pts/m2 in Sweden, Switzerland, Denmark, Finland, Spain. Densities similar to those adopted in the described project are found in German projects. In the Netherlands, a density of 10 pts/m2 is adopted [63, 64]. ISOK created a DTM from a LiDAR point cloud. However, this data structure leads to generalization, which means it cannot achieve the same 1-meter square accuracy as the original point cloud. The DTM derived from this data features a 1.0 m mesh size grid structure and an average height inaccuracy of 0.15 m for paved surfaces. The mesh density for DSM for Standard II is 0.5 m [14]. Blind spot minimization is ensured by two perpendicular mensurations; nonetheless, high density is crucial for replicating significant anthropogenic and small-scale natural landforms. The National Geoportal [65] provides open access to DSM data through its resources, in compliance with the INSPIRE [31] directive and Polish law [66]. The data used in the article is from the year 2023.

### Point cloud

A point cloud is a collection of points in three dimensions, each of them characterised by three coordinates: *x*, *y*, *z*, along with possibly additional properties. These additional parameters may include the colours represented by the RGB components, the point class indicating the type of element the point belongs to, and other factors required for subsequent processing. The number of points in a given region may vary considerably, depending upon the requisite specifications, the available computing space, and the scanning technology chosen. The number of points mostly determines the precision of a point cloud’s mapping of an item. Regretfully, the processing operations associated with a higher point count likewise become more challenging. In the research for the article, a cloud with a density of 6–12 points per square meter was employed. There is considerable flexibility regarding the format in which a cloud may be saved. The LAZ file format, utilized in this paper, is currently the most common. As a compressed version of the .las file, it facilitates speed, ease of transfer, and viewing of data [67].

#### Classification of points in the cloud

The point class may assign appropriate properties to each cloud point and associate it with the object from which the LiDAR radiation beam was reflected [68]. In the context of point cloud generation using LiDAR technology, a point class may be defined as a category or set of properties typically associated with the terrain or its cover objects [69]. The point classes are described in the Table 1.

**Table 1:** Point cloud classes.

Class	Description
Created, never classified	points created, never classified.
Unclassified	points never classified
Ground	points located on the ground
Low Vegetation	points representing low vegetation
Medium Vegetation	points representing medium Vegetation
High Vegetation	points representing high vegetation
Building	points representing buildings
Low Point (noise)	noise points (erroneous points)
Model Key-point (mass point)	key points of the model (mass points)
Water	points representing areas under water

The points were automatically classified using a machine learning algorithm designed to group data based on specific features. In this case, the algorithm utilized sample data to assign points to particular groups. The classification process was based on the analysis of the points’ color and height and was conducted by ISOK.

**Fig 1:**
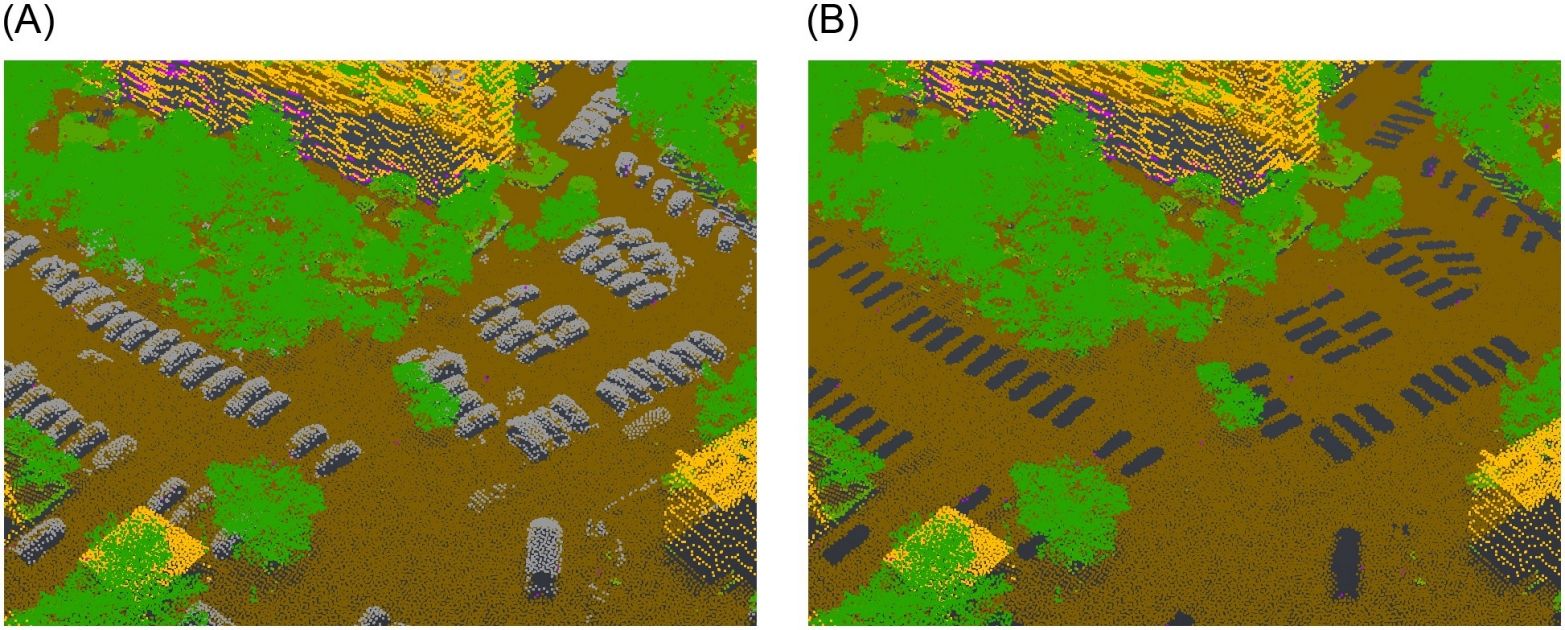
Comparison of a classified point cloud with the vehicles class included (a) and excluded (b). (a) Point cloud with vehicles. (b) Point cloud without vehicles.

Classifying the data is beneficial in subsequent analysis as it facilitates the identification of specific features and objects and allows for filtering. In the context of the presented research, classification proved to be an invaluable tool in the removal of transient elements like individuals and vehicles (Fig 1).

### Viewshed

The term “viewshed” is defined as a graphical representation of areas visible and invisible from a specific position in space, described by coordinates (*x*,*y*,*z*) [70]. The term itself emerged several decades ago [71, 72]. The graph shows binary data regarding which regions are visible from a specific point and which are hidden by surrounding topography or buildings. Viewsheds have a wide range of applications. They serve as the foundation for evaluating the extent of the visual impact of new investments on the surrounding area and are employed in expert judgments on changes in land development. They enable the comparison and assessment of different urbanization scenarios and the identification of locations with the best scenic values.

Visibility within the urban tissue, particularly concerning highly attractive objects, directly correlates with the area’s value when considering various investment scenarios [73]. High scenic values of a place indicated by the visibility range may result in:

1. **Increase in property value**: Areas with better visibility are frequently more in demand, which can raise their commercial value.
2. **Increase the importance of an area**: An area’s prestige can be enhanced by attracting more people and investments through increased visibility.
3. **Sightseeing Protection**: Areas with prominent views could be protected, which would raise the significance and worth of the surroundings.

In contrast to the two-level, black-and-white viewshed, the visibility map presents information in grayscale [11]. It might be a composite of multiple viewsheds, denoting locations where multiple visually appealing objects are simultaneously visible. It can also display an extensive object’s visibility, such as a mountain range. The degree of attractiveness of the view from that place is reflected in the brightness level of each pixel on this map.

Two examples of data that can be used to produce a viewshed are a digital surface model and a point cloud. The subsequent section describes the process for developing a viewshed using information gathered from these two sources.

#### Generating viewsheds based on point clouds

In order to generate a viewshed from a point cloud data set, a surface must first be constructed. Among the various types of surface, the most commonly used are DEM (Digital Elevation Model) due to the widespread use of data registration in a regular grid and TIN (Triangulated Irregular Network) [70, 74]. This approach enables more detailed representation of the landforms. The topography is created from a point cloud by applying Delaunay’s algorithm [75], which connects points into triangles to create a three-dimensional representation of the terrain. Subsequently, an observation point is positioned at the point from which the viewshed is to be generated. Finally, the scene with all objects is rendered from an orthogonal view, resulting in a two-dimensional image representing the viewshed. The white-marked spaces represent the visible areas, while those marked in black indicate zones that are not visible.

The most challenging aspect of generating a viewshed is the creation of a surface. Given the potential for a vast number of points in the cloud, this process is time-consuming and requires advanced computational techniques, which are not always feasible on a standard computer. Consequently, various methods are employed to reduce the total number of points in the cloud, or to split the point cloud [68] to split the point cloud into components, to generate smaller viewsheds with each of these subsequently merged to generate the final viewshed. The paper [76] describes the aforementioned solutions. In our study, we considered a method for reducing points in the point cloud based on the minimum distance between points, set to 20 cm. However, we decided to use the original input data to check for the best accuracy compared to the DSM.

#### Generating viewsheds based on DSM

The process of creating viewsheds can be significantly accelerated and facilitated by omitting the surface generation step. This solution is employed in the QGIS program with the Viewshed Analysis plug-in installed [77]. Thanks to the method used in QGIS, there is no need to create a TIN mesh, which is complex and requires significant computational effort. Additionally, rendering the scene with lighting and surfaces is not required, which leads to a reduction in processing time. It solely relies on raster data from DSM, where a pixel’s value represents the height of a certain point in the terrain. The method starts with a given observation point, finds lines of sight, and generates a binary viewshed from that point on. The application of this method enables the identification of visible and invisible areas to be completed in a more expedient manner. Professionals engaged in the analysis of geospatial data frequently utilise QGIS, as it provides a comprehensive array of tools for data analysis.

### Analysis methodology

A case study methodology was employed in the conduct of the research. Each study was supported by a series of analyses and graphs that provide an in-depth understanding of visibility in urban areas. The methods employed in the studies conducted were of a detailed and multifaceted nature, comprising a number of distinct elements.

1. **Viewshed on digital surface model:** A viewshed was developed for each of the study points based on data from the terrain model. This visualisation enables to identify the visible and obscured areas of the area in question.
2. **Viewshed on a point cloud:** A visibility analysis was conducted using a point cloud, with the objective of obtaining more detailed visibility data in the areas under study.
3. **Visible pixels ratio:** In order to obtain an accurate quantitative assessment of visibility, a ratio of visible pixels to total pixels was calculated in the surveyed areas.
4. **Superimposed viewsheds:** The viewsheds obtained at steps 1 and 2 were superimposed according to the coordinate system references, allowing for a comparison and analysis of the differences between them on a single raster.
5. **Absolute difference:** The absolute difference between the images obtained at step 4 was calculated in order to facilitate a comparison of the viewsheds based on DSM and PC. This comparison revealed areas that were visible in only one of the graphs.
6. **Subtraction:** A statistical analysis of the visible pixels revealed that DSM charts contain a greater number of visible pixels than PC charts. Consequently, the PC charts were subtracted from the DSM charts in order to ascertain the extent of the differences between the two.
7. **Hypsometry maps:** Hypsometry maps were generated to illustrate the discrepancies in elevation between the DSM and the point cloud data. Hypsometry maps depict the elevation variations across the terrain, highlighting the areas in which the DSM and point cloud data diverge. These maps offer insights into the discrepancies in elevation measurements and assist in the understanding of terrain morphology and topographic features.

**Fig 2:**
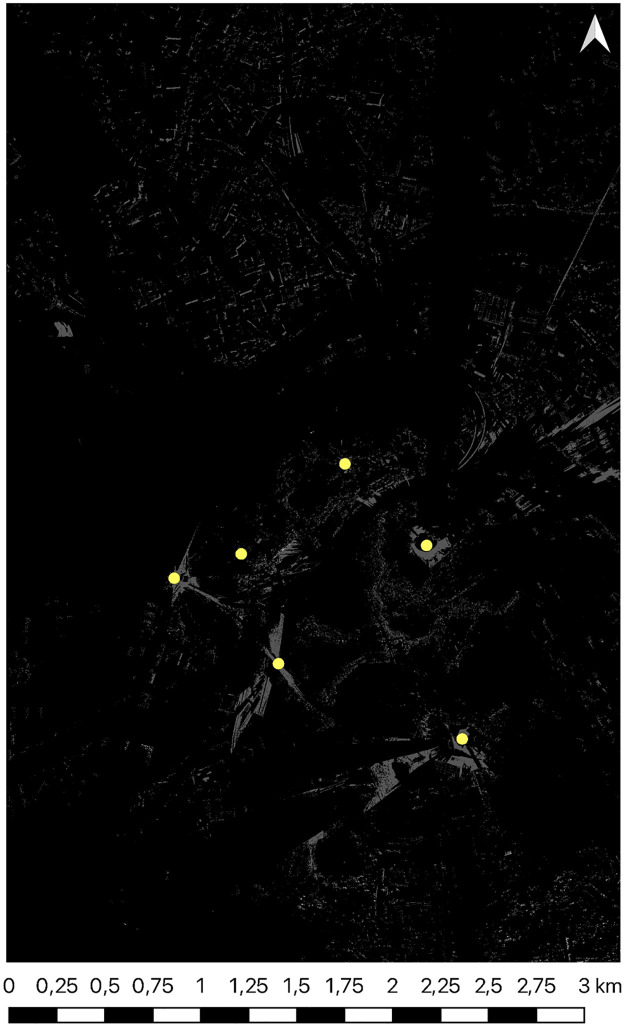
Visibility map generated for all points from Table 2 on DSM input.

The primary impediments to further research were identified as the noise present in the data under study. As a strategy to mitigate this issue, objects classified as vehicles and people were excluded from the point cloud. Filtering the source of the DSM is not possible because the data only stores information about the location of the point and its height. Additionally, difficulties arose when attempting to generate viewsheds on the point cloud for large areas. However, these operations were relatively straightforward when working with DSM data (Fig 2).

## Results

**Fig 3:**
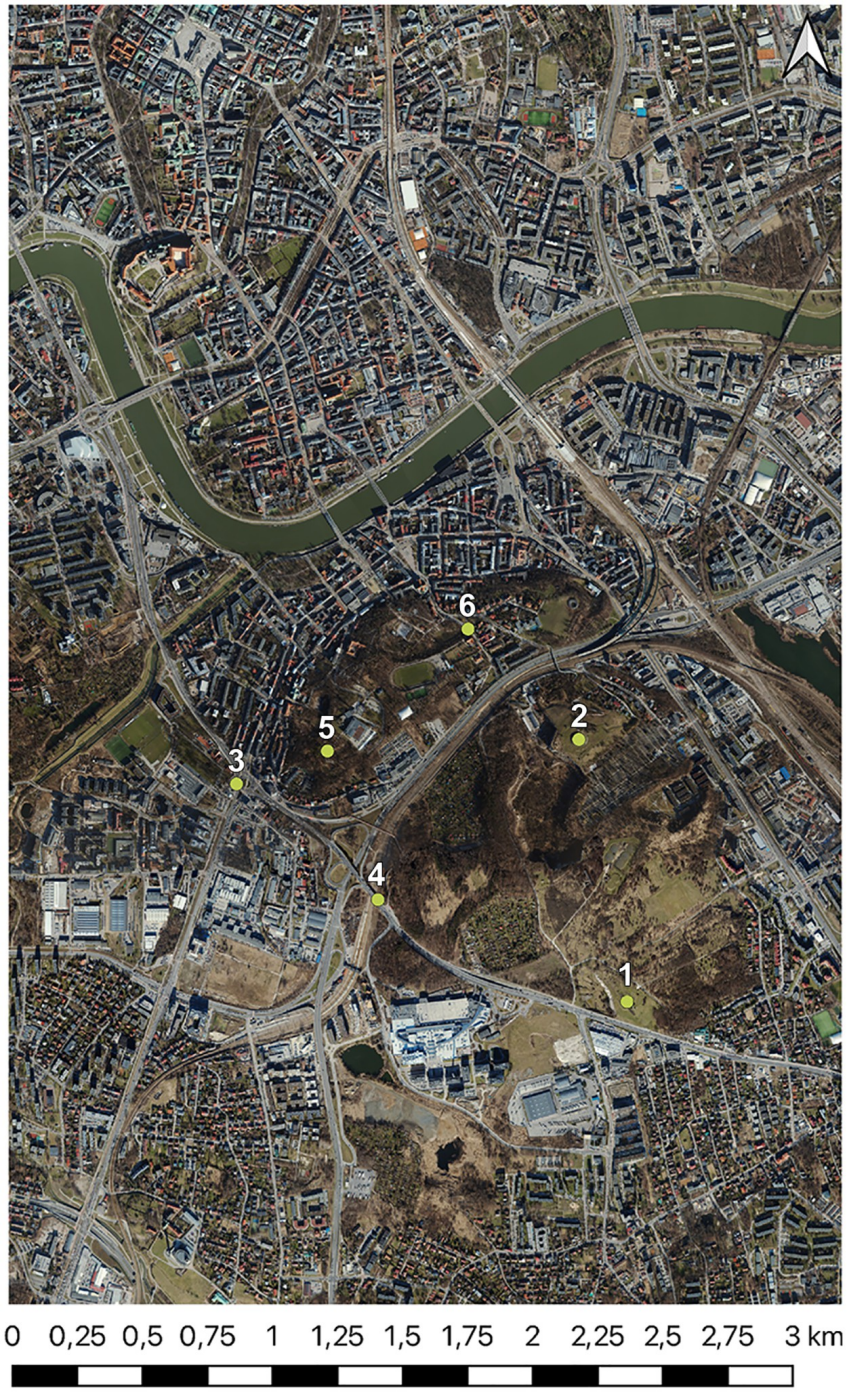
Orthophotomap of the analyzed area with marked points from Table 2. Satellite image source: Geoportal.

For our study, we choose six key points located in urban space (Fig 3) that represent a variety of topographical and urban conditions (Table 2). Each of the given points was carefully selected to allow for a detailed analysis of visibility in their surroundings. Geographical coordinates are given in the WGS84 system.

**Table 2:** Points used for calculating viewsheds.

No.	Point name	Latitude	Longitude	Description
1	Monument	50.029013	19.961229	View from a small hill limited by land cover elements
2	Krakus Mound	50.038056	19.958445	View from the vantage point indicated in the planning documents
3	Mateczny	50.036354	19.940109	View from the transport hub
4	Kamińskiego	50.032418	19.947787	View from the road viaduct on the railway line
5	Krzemionki	50.037537	19.944963	View from a hill covered with forest
6	Parkowa	50.041823	19.952418	View from a small square in a suburban district

**Fig 4:**
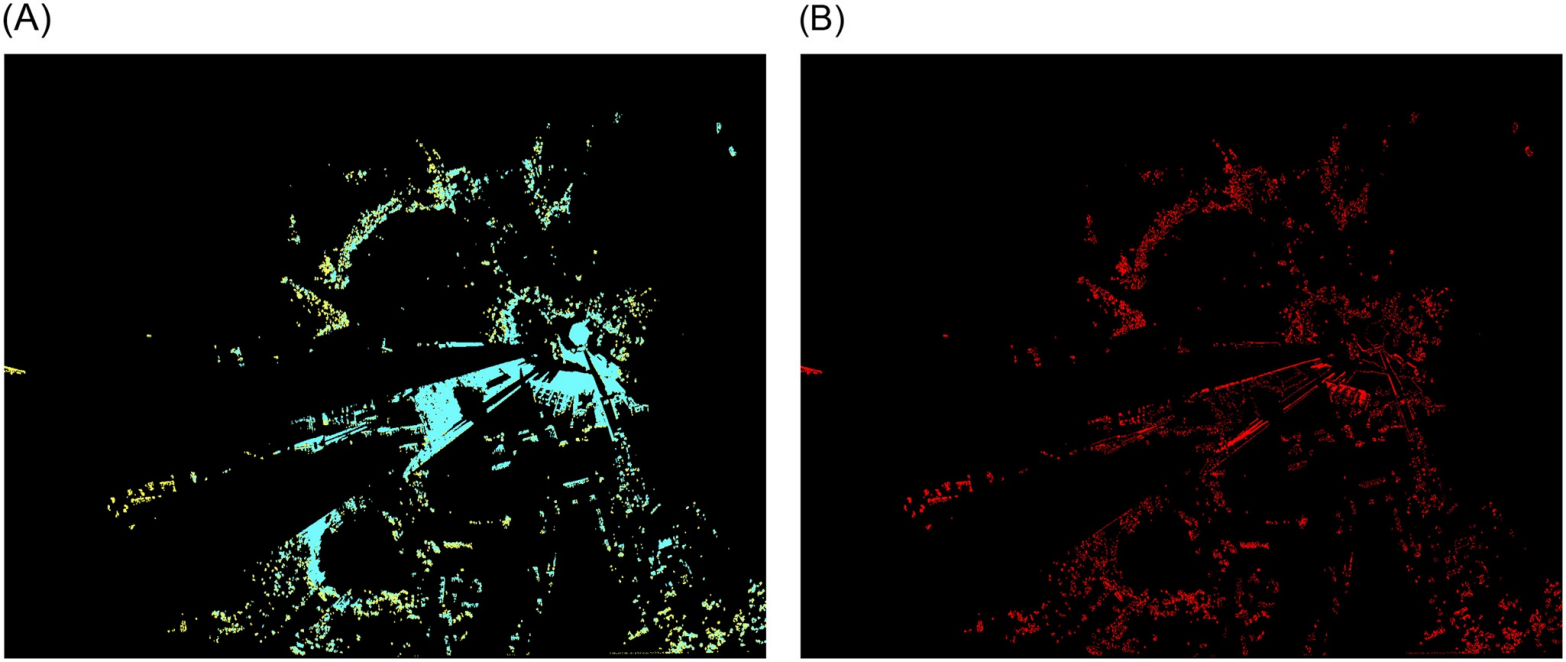
Extended analysed area for the Monument to the Victims of Fascism. Yellow—viewshed based on DSM, cyan—viewshed based on point cloud. (a) imposed viewshed. (b) difference.

**Fig 5:**
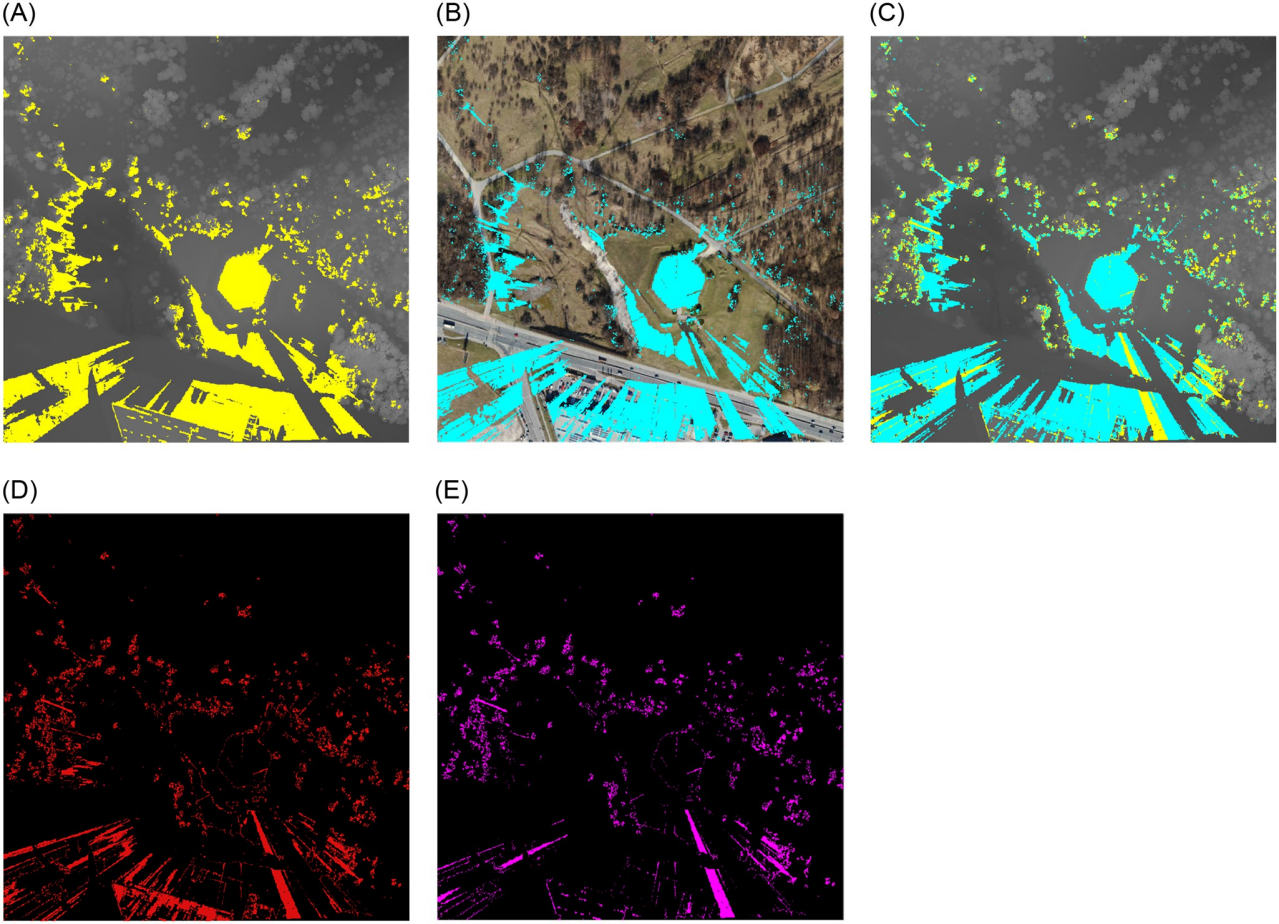
Comparision of viewsheds for the Monument to the Victims of Fascism in Krakow. Satellite image source: Geoportal. (a) DSM input. (b) Point Cloud input. (c) DSM and PC. (d) Difference. (e) Subtraction.

For the initial viewsheds from point 1 (Monument), two area sizes were taken into account. The first studied area covers 3.579 km² (2022.00 m x 1770.11 m) with each pixel representing an actual length of 1.6 meters (Fig 4), and the second area (554.80 m x 587.06 m) of 0.326 km² is discernible at a pixel corresponding to an area of 0.6 m x 0.6 m (Fig 5).

The objective was to gain a comprehensive understanding of the data, which was achieved by examining data produced by two distinct techniques: point cloud and digital surface model.

For data covering a larger area (first area for point 1) no discernible differences were identified between the two models at this spatial resolution. Consequently, it was determined that a reduction in the extent of the analysis area for each point and an increase in the spatial resolution would be beneficial.

Following a series of tests, it became evident that the differences in spatial resolution (second area for point 1) are discernible at a pixel corresponding to an area of 0.6 m x 0.6 m (Fig 5). To gain further insight into the variability of visibility in urban environments, we established a corresponding range (within a radius of 150 m to 200 m from the visibility point) for each point. This was intended to demonstrate the differences in detail and to illustrate the variability of visibility depending on location and topographic surroundings for different conditions.

### Point 1—Monument to the Victims of Fascism

Point 1 (Table 2—Monument) is situated along the edge of the old rampart of the Kraków fortification from the 19th century. Encircled by a moat, the rampart’s summit resembles a hexagon. The 1.5 m recess that makes up the rampart’s interior is apparent from the crown. A limestone hill was excavated in the western section, and the earthwork was built atop it. This was the location of the German concentration camp KL Płaszów during World War II. In commemoration of the victims, a stone memorial was thus built upon the rampart [78, 79]. This structure is located close to point 1, at a distance of 6 metres, and acts as an object that restricts the view from that point. Additionally, three little trees nearby block the sight. Tall foliage to the east, north, and west obscures the view farther away. A broad connection artery lies to the south of the point, behind which there are various urban buildings. Nevertheless, the rampart’s apex is elevated enough to view the lower buildings’ roof surfaces.

A comparative analysis of the two viewsheds generated using the point cloud (Fig 5b) and the digital surface model (Fig 5a) from the Monument to the Victims of Fascism revealed significant differences in the detection of visible areas. The visible area in the viewshed obtained from the point cloud exhibited a greater number of radial splits. The more generalised DSM model excluded certain objects, which is why they were omitted. The radiality of the views emanating from a single point is a consequence of their spread. From a distance of approximately six metres from the vantage point, the Monument to the Victims of Fascism creates an empty area in the PC viewshed but not in the DSM viewshed. Furthermore, the effect of the trees growing on the hill, which obscure the walls, is observed. This leads to the conclusion that the viewshed created on the point cloud is more precise, as it includes more details that are significant at close range. In the case of a small observation area, with no possibility of movement to circumvent an obstruction, this becomes a significant issue. This does not apply in this instance, but it is a common occurrence in urban landscapes.

The visible area is more compact and less fragmented in the viewshed acquired on DSM. In areas where the PC model’s surface is visible, the DSM model’s surface creates impediments, as evidenced by the visual analysis (Fig 5), pixel counts (Table 5), (d) Difference and (e) Subtraction. In comparison to the (e) Subtraction map, it is noticeable that the (d) Difference map covers a larger area. It can be expected that these graphs will match, given that the PC model is more realistic and includes items that block the view at close range. The distinction between them can be attributed to the DSM model’s simplification; for instance, certain locations are visible in the PC model but not in the DSM, and vice versa.

**Fig 6:**
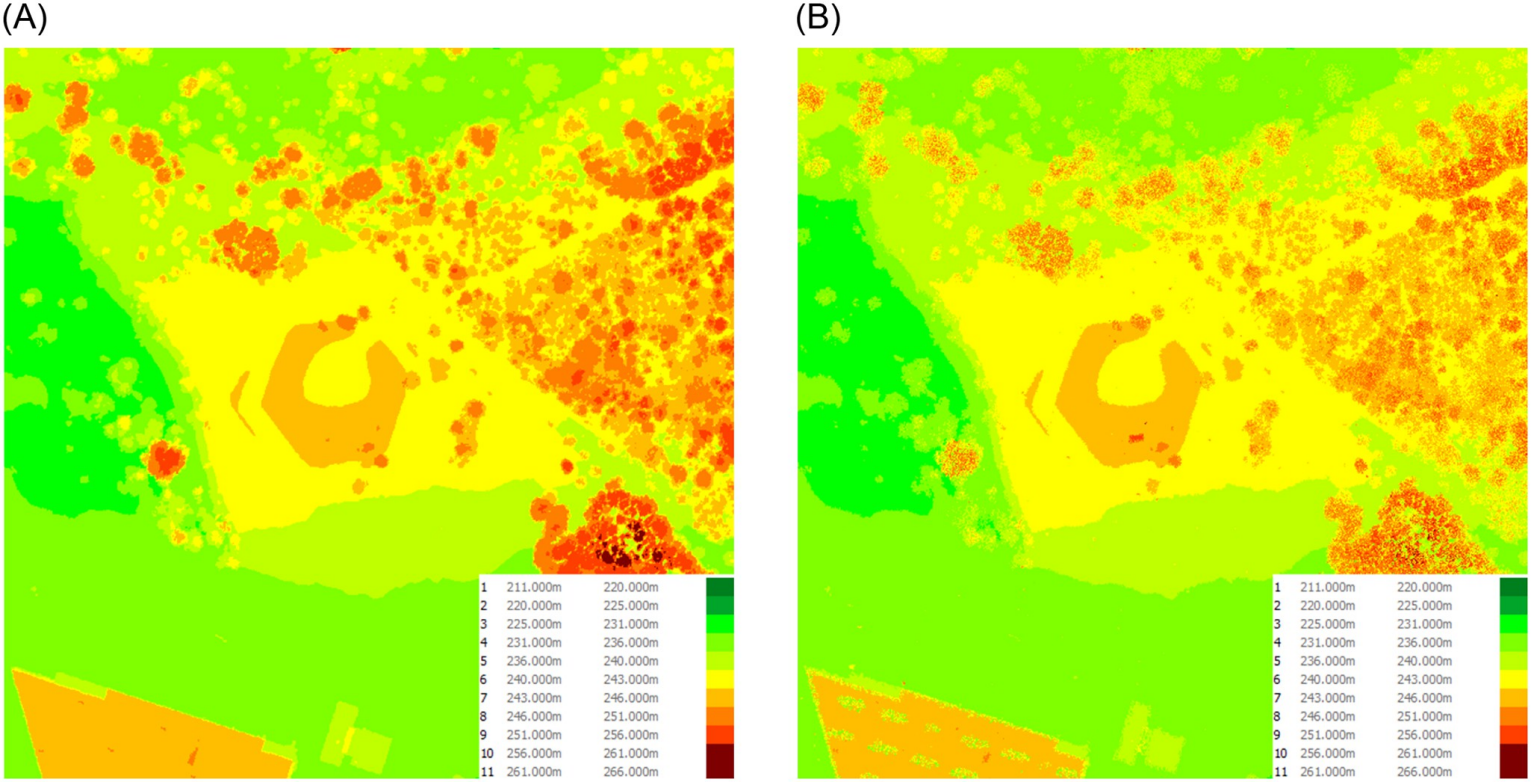
Comparison of hypsometric diagrams for the DSM (a) and the Point Cloud (b) of the depicted slice for the Monument to the Victims of Fascism. (a) DSM Hypsometry map. (b) TIN Hypsometry map based on PC.

In our study, we compare additionaly two hypsometry maps (Fig 6) for the selected point. One map was generated from a digital surface model, while the other was generated from point cloud data. The analysis focuses on the level of detail, surface representation, and statistical differences between the two maps, which are crucial for accurate viewshed analysis.

The hypsometry map derived from point cloud data exhibits greater detail and distinctiveness, including the representation of individual trees, due to the higher resolution and accuracy of point cloud measurements. In contrast, the DSM-based map provides a more generalized representation of the surface, with a tendency to smooth over many smaller features. This distinction is evident when examining specific points on the map, where the point cloud data captures more surface irregularities compared to the smoother DSM representation.

### Point 2—Krakus Mound

**Fig 7:**
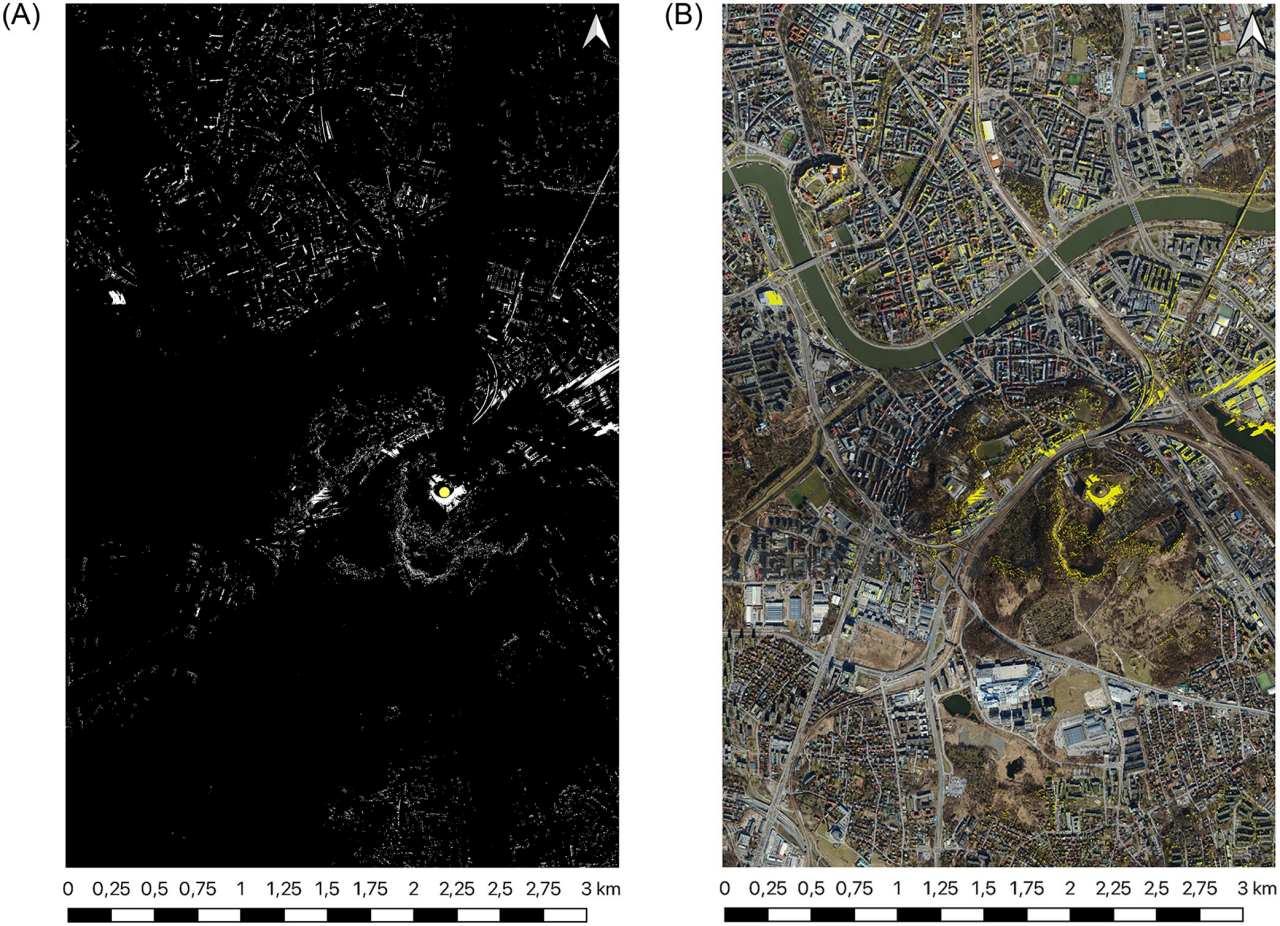
Visibility map generated for Krakus Mound (point 2) (a) based on DSM input for the entire study area (b) layered on sattelite view Satellite image source: Geoportal.

One of the city’s most exposed vantage points is the Krakus Mound (Fig 7, Table 2—point number 2). It is situated on a barrow that dates back to the early Middle Ages. Situated at an elevation of 271 meters above sea level, the mound is underdeveloped and slightly covered with vegetation. Its base lies approximately 40 meters away from a steep quarry slope that is 42 meters deep. The mound has a diameter of 57 meters at the base, a height of 16 meters from the base, and a flat top of 8 meters. Currently, there are no obstructions to the view at close range. The only reason the mound’s slopes and base are hidden from view is because of its top flattening. Furthermore, the view is also naturally restricted by the quarry slope.

The DEM model’s generalisation is the primary cause of the differences between viewsheds. The observed numerical differences in viewshed precision (Table 3) support the idea that the generalization inherent in DEMs leads to less precise viewshed outcomes than the more detailed LiDAR-based viewsheds. This correlation between the generalization level and the discrepancy extent underscores the impact of DEM generalization on viewshed accuracy. Additionally, in urban areas with complex building layouts (points 1 and 3 from the author’s example, Table 2), the viewshed from a DEM shows certain areas as visible when, in reality, they are obscured by structures that the generalized DEM fails to represent accurately.

**Table 3:** Difference and subraction for viewshed analysis based on DEM and point cloud.

		DIFFERENCE PC AND DEM		SUBTRACTION PC AND DEM	
No.	Point name	Number of pixels	% of pixels	Number of pixels	% of pixels
1	Monument	41 048	5.94%	24 511	3.55%
2	Krakus Mound	37 163	4.94%	29 291	3.89%
3	Mateczny	14 976	1.85%	11 358	1.40%
4	Kaminskiego	16 967	1.42%	12 566	1.05%
5	Krzemionki	650	3.32%	542	2.77%
6	Parkowa	1 224	3.12%	908	2.31%

**Fig 8:**
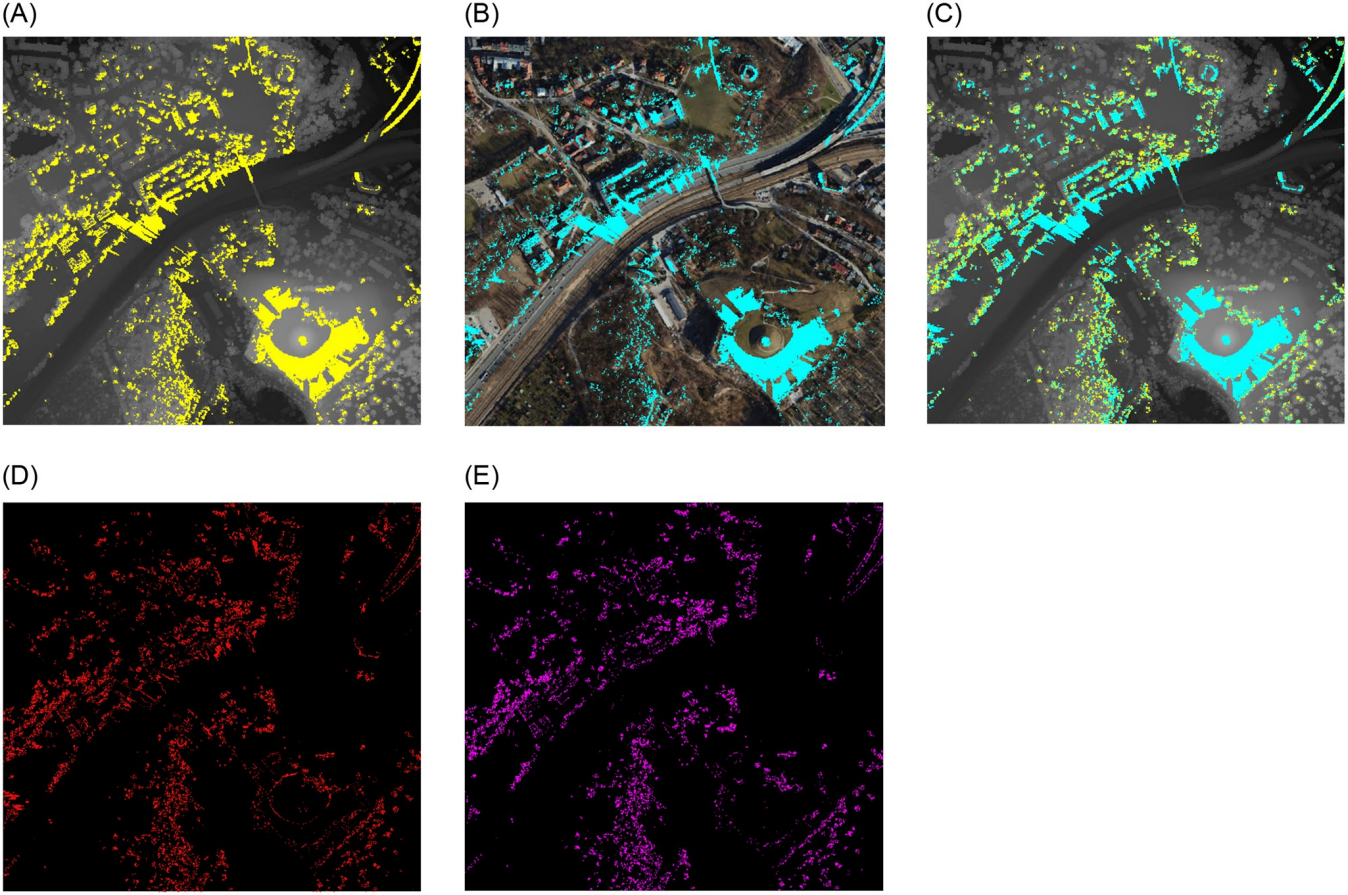
Comparision of viewsheds for Krakus Mound. Satellite image source: Geoportal. (a) DSM input. (b) Point Cloud input. (c) DSM and PC. (d) Difference. (e) Subtraction.

There are no radial splits in the viewshed surface, since no objects nearby block the view from the position atop the mound. A small number of bushes and trees that grow beneath the mound provide effects that are similar in shape on both graphs (Fig 8).

Upon examination of the information presented in Table 3, it becomes evident that the number of pixels differentiating the two graphs is relatively large. This is due to the fact that the viewpoint is situated at a considerable height above the treetops. As a result of the grid extending to the highest points of the cloud, tree tops are more strongly represented in the DSM.

**Fig 9:**
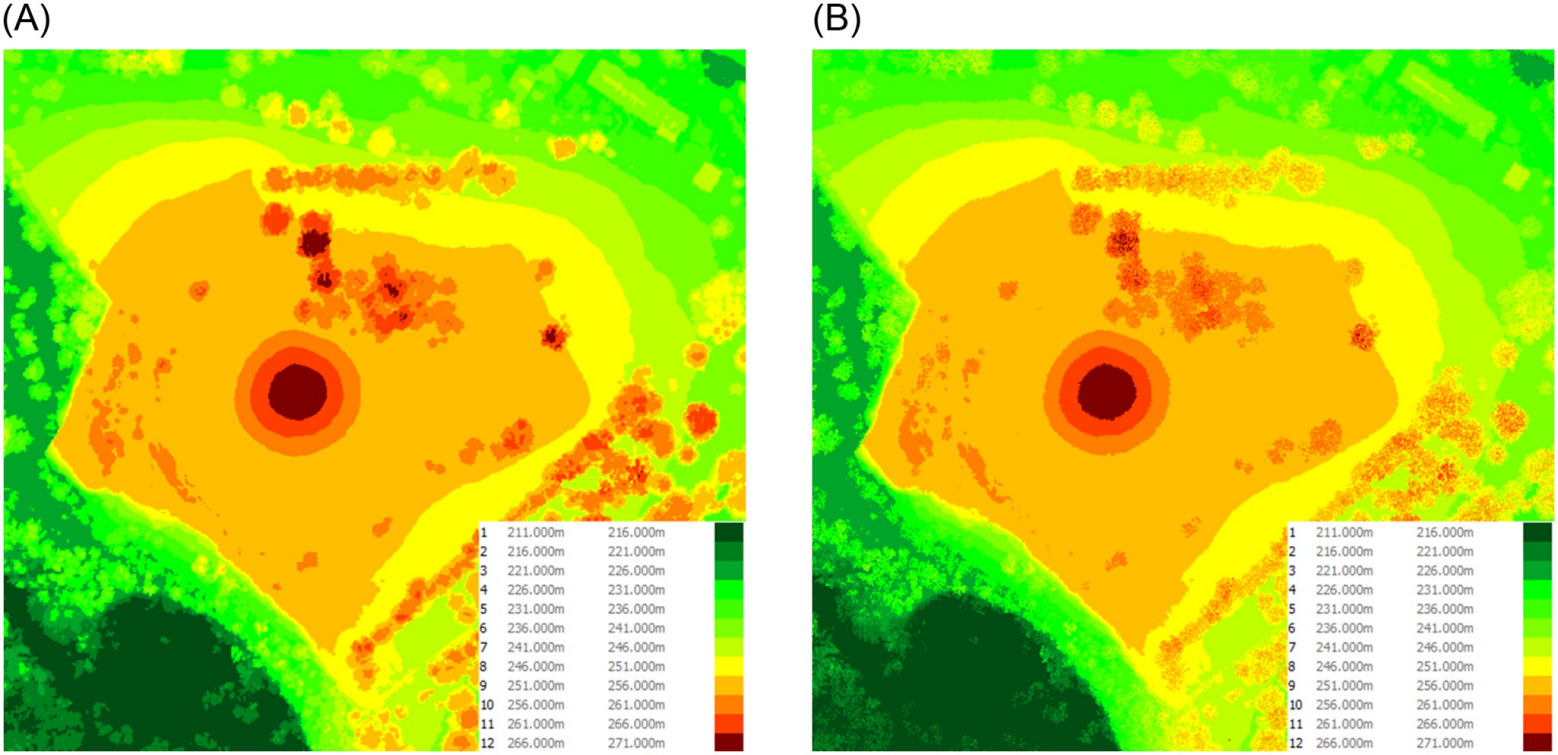
Comparison of hypsometric diagrams for the DSM (a) and the Point Cloud (b) of the depicted slice for the Krakus Mound. (a) DSM Hypsometry map. (b) TIN Hypsometry map based on PC.

Both models have a fairly similar mound hypsometry. The hill and mound are shaped with subtle changes in surface slope rather than having sharp edges. The models only differ in how the former quarry’s edge is depicted. The surface of the mound and the area around it are identical in shape (Fig 9).

### Point 3—Mateczny Roundabout

**Fig 10:**
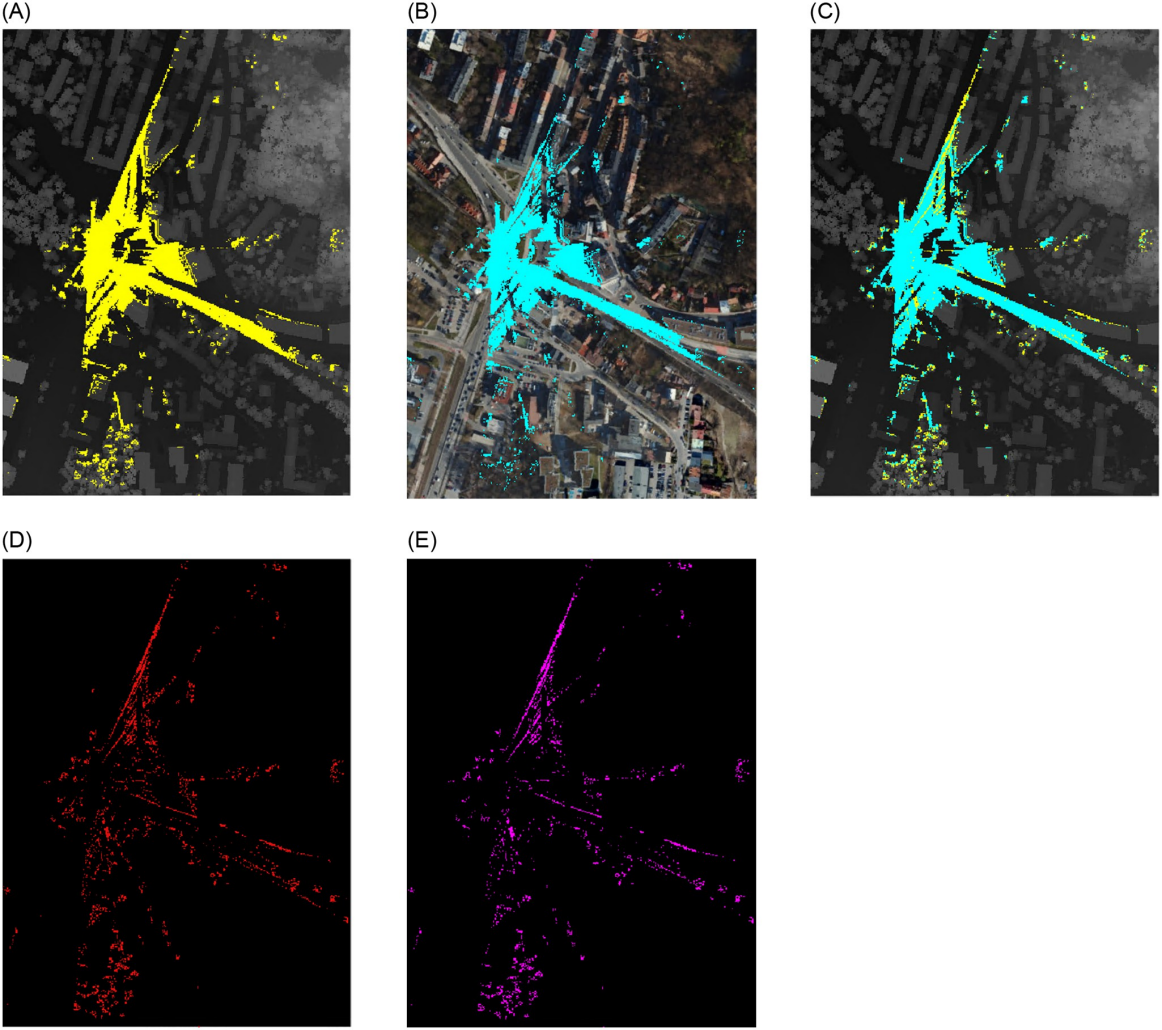
Comparision of viewshed graphs for Mateczny Roundabout. Satellite image source: Geoportal. (a) DSM input. (b) Point Cloud input. (c) DSM and PC. (d) Difference. (e) Subtraction.

The next point, number 3 (Table 2) at Mateczny Rondabout is situated at a lower elevation than the preceding location. It is surrounded by high-density urban development and located in an area with heavy bicycle and pedestrian traffic. It is situated close to Spa Park and a small square. The large buildings to the north, east, and south, as well as the tall trees and park facilities to the west, obstruct the view from this location. It does, however, provide viewing openings along broad street arteries and towards the roundabout (Fig 10).

A detailed analysis of the viewsheds depicted in Fig 10 reveals a number of variations that can be attributed to the presence of a variety of smaller objects, including traffic lights, street lighting and low-level vegetation, which obstruct the view in the PC model. The presence of gaps in the chart, which stretch in a radial direction from the viewpoint, serves to highlight these obstructions. This confirms the PC model’s higher level of detail. However, in this particular case, the process of generalisation employed in DSM has resulted in a marginal increase in the PC viewshed, as reflected in the relatively low number of pixels distinguishing the graphs in Fig 10(d) “Difference” from Fig 10(e) “Subtraction”.

**Fig 11:**
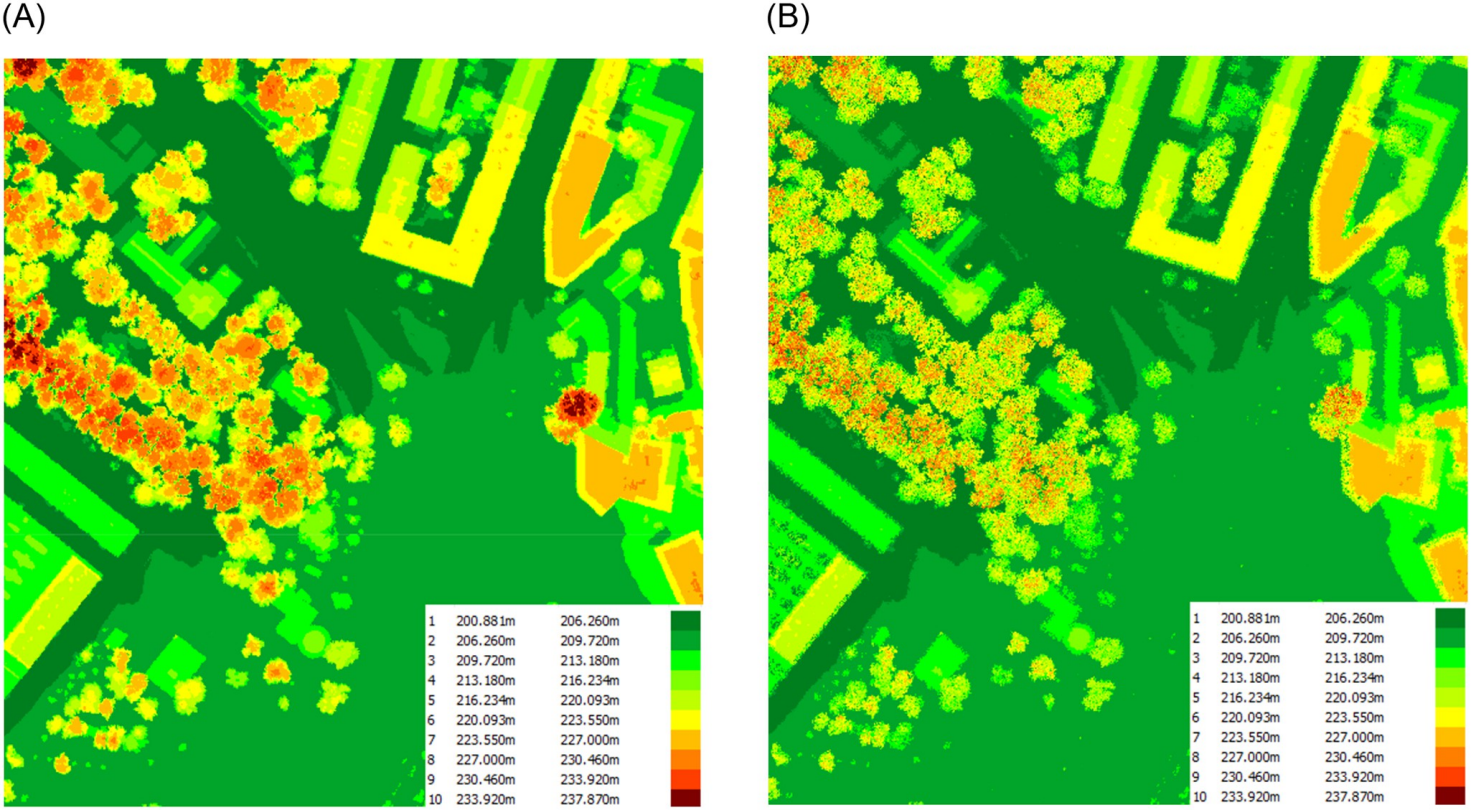
Comparison of hypsometric diagrams for the DSM (a) and the Point Cloud (b) of the depicted slice for the Mateczny Roundabout. (a) DSM hypsometry map. (b) TIN Hypsometry map based on PC.

A comparison of the hypsometric maps created on the PC and DSM models (Fig 11) reveals that, as in the preceding case, the PC map is more detailed. However, what stands out in this case is the curvature of the lines that depict the building edges, a feature absent from the DSM model. This is because the point cloud was triangulated to create an occluding surface.

### Point 4—Kaminskiego Street

**Fig 12:**
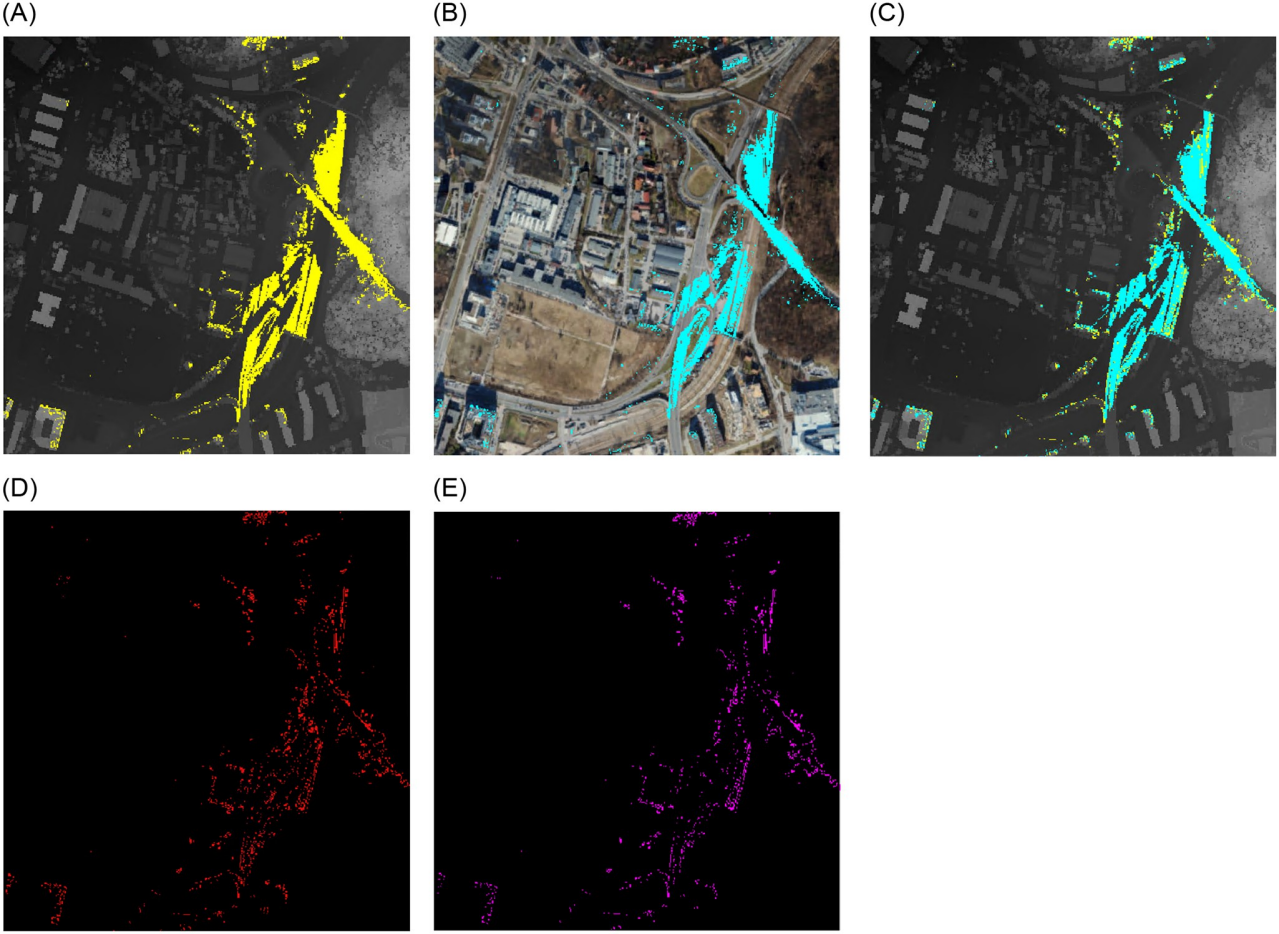
Comparision of viewshed graphs for Kaminskiego. Satellite image source: Geoportal. (a) DSM input. (b) Point Cloud input. (c) DSM and PC. (d) Difference. (e) Subtraction.

The subsequent viewpoint can be found in the vicinity of the entrance to the viaduct spanning the railroad tracks on Kaminskiego Street. Despite being situated in proximity to an area densely forested, the view from this vantage point encompasses a bustling communication artery along with lateral views of the adjacent railway line and related technical infrastructure, imparting a metropolitan character to the locale (Fig 12). The surrounding area is perceived as an industrial region due to the high concentration of technical facilities.

The gaps in the PC graph surface that extend radially from the viewpoint are an aspect that distinguishes the two viewsheds. Fig 12(d) and 12(e) illustrate this phenomenon. The appearance of these gaps is a consequence of the presence of road infrastructure components, including street lights, traffic signals, and road signage. Nevertheless, these features are not included in the DSM model, which represents a limitation of the point cloud’s accuracy.

The data on viewshed differences based on DSM and PC presented in Table 3 illustrates that the nature of this perspective is comparable to that of point 3—Mateczny Roundabout. Despite being of a different kind, the view is similarly urban. In contrast to points 1 and 2, where the tree crowns take up a larger portion of the picture, there are fewer distinctions between the graphs.

### Point 5—Krzemionki Hill

A further case of analysis is Krzemionki Hill, a recreational area characterised by a high degree of woodland cover (point 5 from Table 2). The dense foliage in the immediate vicinity limits visibility, resulting in an intimate and somewhat restricted view.

**Fig 13:**
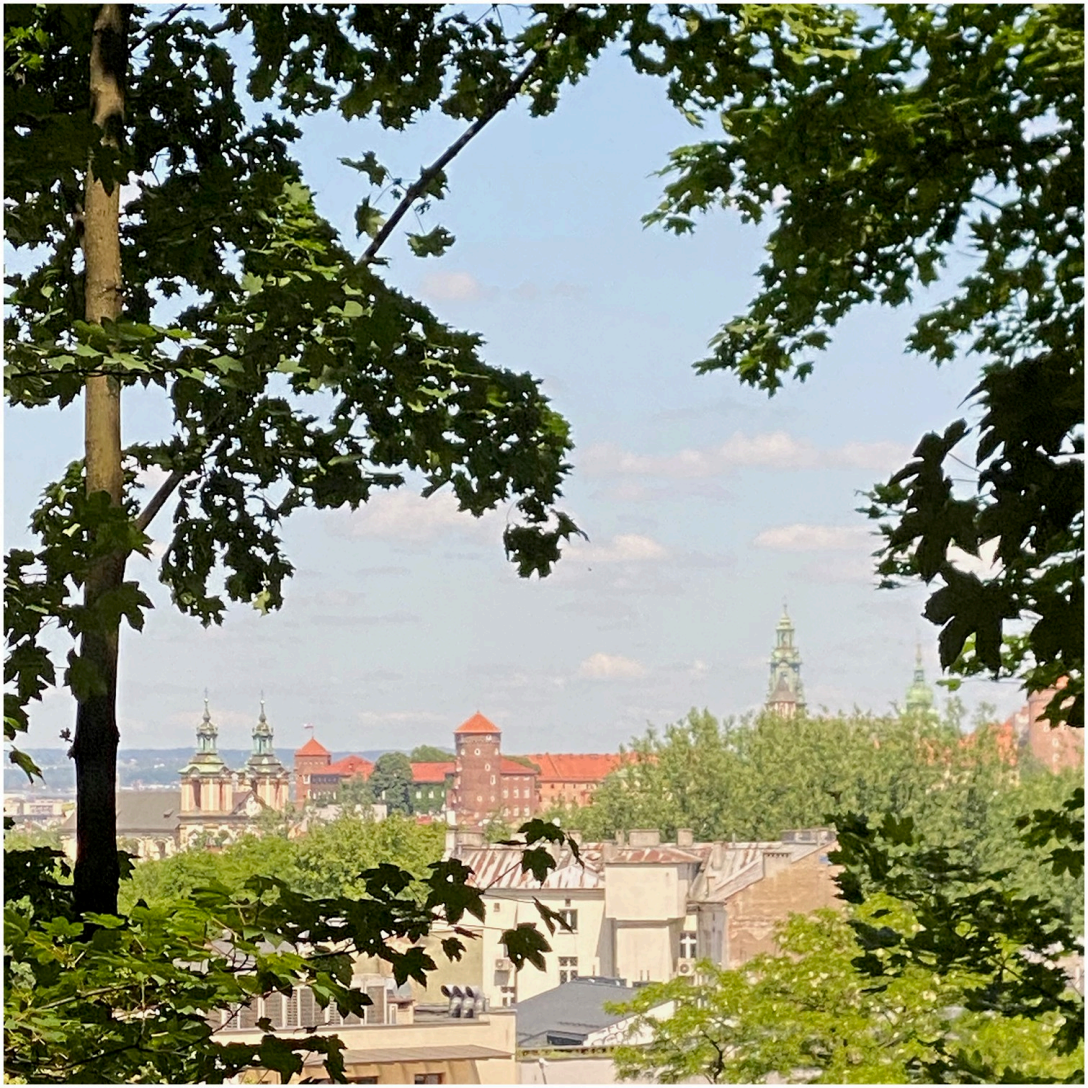
Panorama of the Wawel Royal Castle in Cracow from a point on the Krzemionki Hill (Table 2.—point 5). Image by the author A. Widlak.

The view of Wawel Castle, one of Poland’s most significant landmarks, is not represented in the viewshed. However, it can be observed in situ through the viewing windows between the tree trunks s (Fig 13). This is due to the limitations of the model, which encompasses the highest points without consideration of crevices and cavities beneath tree canopies.

**Fig 14:**
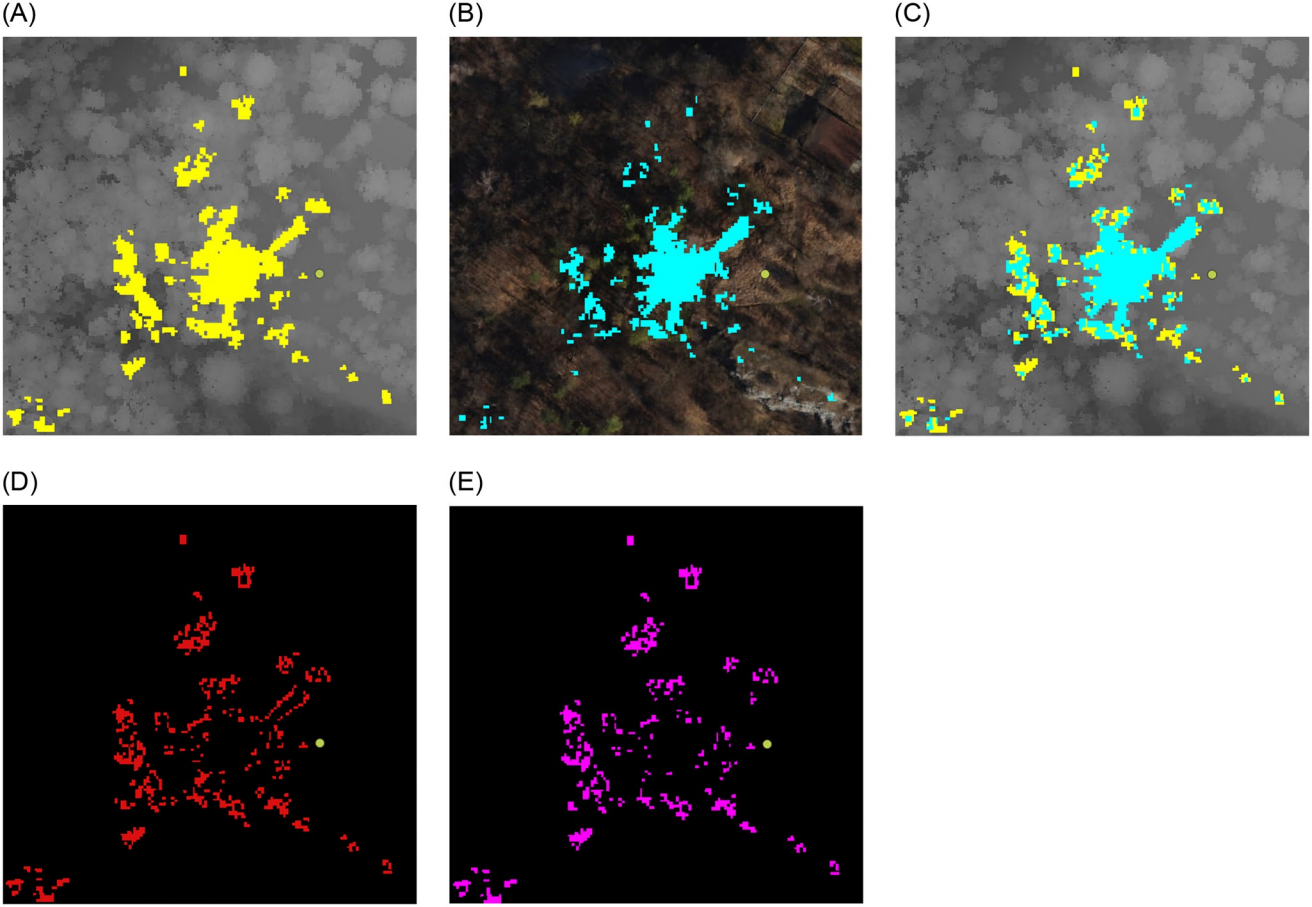
Comparision of viewshed graphs for Krzemionki in Krakow. Satellite image source: Geoportal. (a) DSM input. (b) Point Cloud input. (c) DSM and PC. (d) Difference. (e) Subtraction.

The visible area of the digital surface model is 4.35%, while that of the point cloud is 3.67%. This discrepancy is evident in the viewsheds produced by the two models (Fig 14). The primary cause of this difference is the lesser accuracy of the DSM, which smooths the model and generalises the data in regions with tree cover.

### Point 6—Parkowa Street

The final point (Parkowa from Table 2.) is situated on Parkowa Street, which is used for local transportation. The western boundary of the site is surrounded by Bednarski Park, which was established in the 1800s. The park comprises the conserved remnants of an earthen fortress dating back to the 18th century, which was constructed on the site of a defunct quarry. The area’s geography and extensive tree cover significantly restrict the view in this direction. A triangular plaza with villa structures situated behind it is located to the north of this location. The square is primarily composed of grass. In the distance, the tower of the neo-Gothic church can be seen. The neighbourhood’s single-family residential buildings can be observed from the east and south, interspersed with slightly larger service buildings (kindergarten, convent). The visibility chart extends into the street axis and is essentially restricted to the square area. Due to the high terrain’s slope towards the north, some building rooftops are visible.

**Fig 15:**
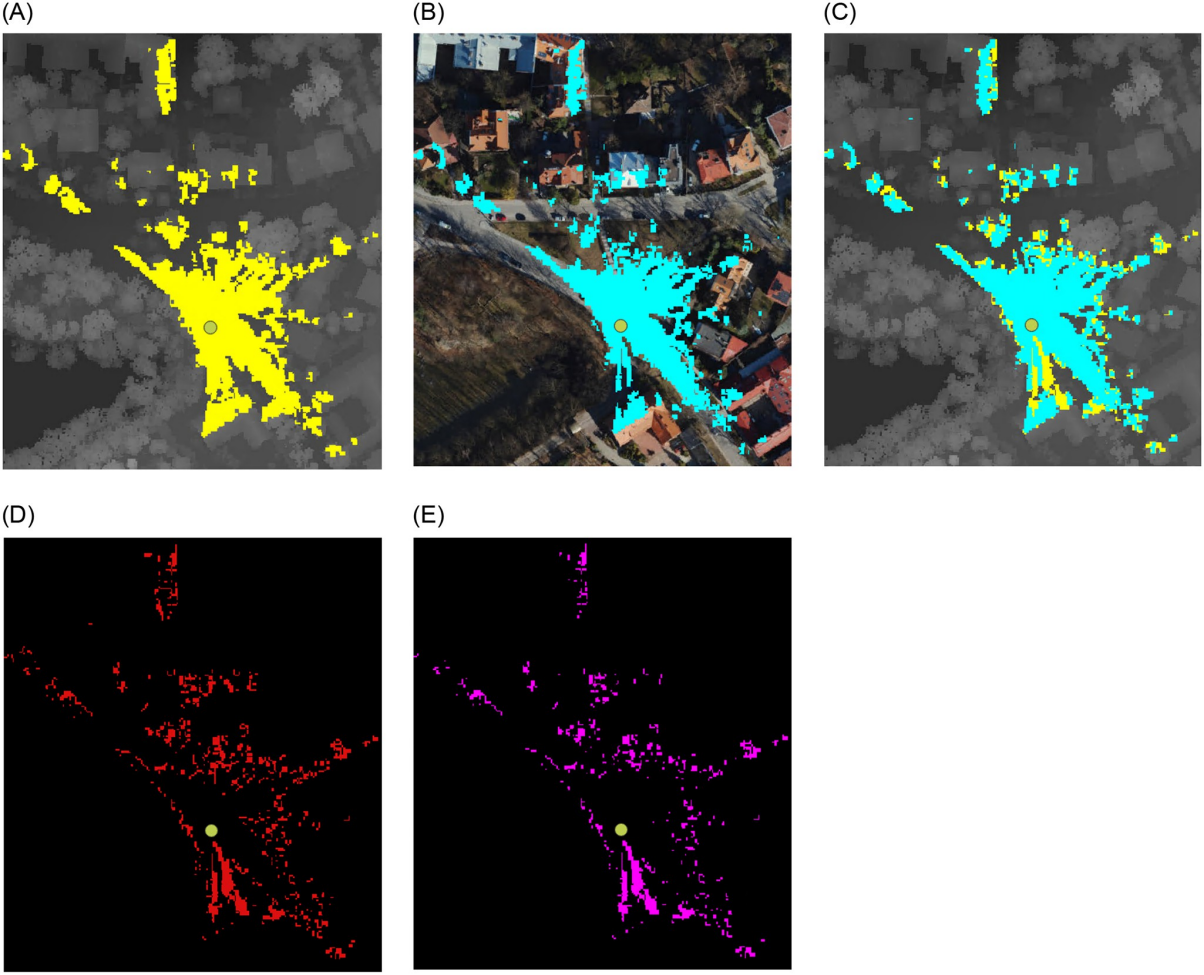
Comparision of viewshed graphs for Parkowa in Krakow. Satellite image source: Geoportal. (a) DSM input. (b) Point Cloud input. (c) DSM and PC. (d) Difference. (e) Subtraction.

The discrepancy between the obtained viewsheds is minimal, amounting to a mere 0.19 percentage points. The area in question is characterised by the presence of a park, roadside greenery, and several scattered buildings in its immediate vicinity. In accordance with the observations made in other instances, the area covered by the DSM viewshed is slightly larger than that covered by the PC. The latter is of a higher level of detail due to greater surface accuracy, a fact that is particularly apparent in the southern sector of the viewpoint. The spaces that open up between the buildings and trees are illustrated in Fig 15).

A series of viewshed comparisons have been conducted at six locations within a major urban area. The research involved the selection of points in a number of different urban zones, with the aim of providing a comprehensive overview of the surrounding environment from each location. The article presents a detailed analysis of six distinct locations, each of which offers a unique perspective on the urban landscape:

1. A small hill with an open view
2. A typical tourist viewpoint
3. A point on the square among the metropolitan buildings
4. A metropolitan district with a dominant share of transport and industrial infrastructure
5. A densely wooded area with a view strongly limited by tall greenery
6. A district with low-intensity development

The statistics presented in Table 4 prove the views’ diversity. The view range is indicated by the value in the “Total Pixels” column. This is the number of pixels contained within the map rectangle defined by the furthest points visible from a given location in each of the four basic directions (north, south, east, west). The column designated “Visible pixels” indicates the number of pixels that have been identified as visible by the viewshed generation algorithm. A high number in this column indicates a broad, unobstructed view, while a low number indicates a personal, neighborhood view. The ratio of visible to total pixels is shown by the value “% of Visible pixels”. In this column, views that are open are valued higher, views that are in a large town are valued medium, and views that are intimate are valued lower.

**Table 4:** Result for viewshed analysis based on DSM and point cloud given in pixels.

				DSM		Point Cloud	
No.	Point name	Area km^2^	Total pixels	Visible pixels	% of Visible pixels	Visible pixels	% of Visible pixels
1	Monument	0.249	691 392	99 556	14.40%	91 582	13.25%
2	Krakus Mound	0.271	752 463	81 997	10.90%	60 578	8.05%
3	Mateczny	0.291	808 839	59 876	7.40%	52 136	6.45%
4	Kaminskiego	0.430	1 193 284	60 647	5.08%	52 482	4.40%
5	Krzemionki	0.007	19 591	1 376	7.02%	942	4.81%
6	Parkowa	0.014	39 246	4 432	11.29%	3 840	9.78%

The number of visible pixels in the viewshed created using the point cloud was consistently smaller than the one derived based on the digital surface model in all cases. This discrepancy was largely attributable to the model’s generalisation and the selected visibility computation algorithm, as previously discussed in the analysis of the results for each point. The PC chart layout was more fragmented and included a substantial quantity of details. The graphs incorporated the depressions between trees and small buildings, resulting in the appearance of “view windows”—small areas that are visible—in these locations.

The time required for the computation of viewshed graphs was meticulously measured. The total computation time encompasses the execution required for mesh creation, which was derived from a given point cloud in the case of point cloud (PC) processing.

The computations were performed on a system with the following specifications:

- **Processor:** Intel(R) Core(TM) i9-14900K CPU @ 3.20 GHz
- **Memory:** 64.0 GB (Available: 63.8 GB)
- **Graphics:** Integrated UHD Graphics 770

It’s important to note that the operations were run solely on the processor without the support of GPU processing. This ensures that the measured times accurately represent the performance of the CPU in handling these tasks.

The processing times listed in Table 5 indicate that the creation of viewsheds in a point cloud is a time-consuming process. The objective of this research is to ascertain whether it is advantageous to reduce the processing time by using DSM as input, despite the potential for obtaining less detailed and precise results.

**Table 5:** The time needed to create a viewshed based on the rendering method.

Time	data
98min 52sec	Point Cloud
9min 3sec	DSM

## Discussion

The objective of this study was to identify the most appropriate method for generating accurate viewsheds in an urban landscape. Two methods were evaluated for their suitability for generating viewsheds. Analysing viewsheds based on point clouds, it is possible to see view shadows coming from small elements of space such as street lamps, advertising poles, kiosks and even street signs. The shadows from these elements are not visible on viewsheds created with DEMs. This can be seen even better by analysing the difference and subtraction images on the orthophoto base, where the cause of the shadow can be identified. Based on this observation, point clouds can be recommended for small spatial ranges. For large distances, small elements of space are not important, they are invisible. At the same time, large distances cover large areas and large data sets. Therefore, data generalised in DEM sets can be effectively applied to large areas. The first method is based on digital surface model data and uses raster input. The second method is based on point cloud input and leverages detailed three-dimensional point cloud data obtained from sources such as LiDAR.

The effectiveness of both methods was assessed based on the presented six distinct urban scenarios. Owing to the city’s wide variety of landscape enclosures, the six cases chosen for research are not representative of every possible spatial scenario within the townscape. A broad range of different types of places, surrounded by buildings, vegetation, and components of the transportation and technical infrastructure, were all considered. The experts who conduct landscape studies for cities provided their experience in selecting the examples. Consequently, urban structures were left out, for which analyses provide precise results, making it unnecessary to compare the two methods. A straight park path leading to a palace or a densely built-up city street with a clearly defined viewing axis would be examples of such kinds of spaces. The comparative analysis revealed significant discrepancies in the performance of DSM and PC data for viewshed generation in urban environments.

The quantitative comparison between different methods of viewshed generation is summarized in Table 6.

**Table 6:** Comparison of viewshed generated from point cloud and DSM.

Feature	Point Cloud	DSM
Data Source	Can be filtered based on classification (e.g., buildings, cars, vegetation)	Data source cannot be filtered
Data Accuracy	Accurate, 12 points per m^2^	Less accurate, 4 pixels per m^2^
Data Processing	Heavier processing, impossible for large areas in a short time	Easy to operate on large data areas where accuracy is not crucial
Graph	We can obtain a grayscale graph emphasizing the intensity of visibility areas	We obtain a bitmap graph, visibility information is approximated

The point cloud method provided a higher level of detail and accuracy in the viewshed analysis. With 12 points per square meter, it significantly outperformed DSM, which has only 4 pixels per square meter. This increased accuracy is crucial in urban environments where fine details, such as the precise locations of viewing windows and minor architectural features, affect visibility.

One significant drawback of the point cloud approach is the substantial processing requirement. It is computationally intensive, making it impractical for the rapid analysis of extensive areas. In contrast, DSM is less demanding in terms of processing power, allowing for expeditious analysis over extensive regions, albeit with reduced accuracy.

Nevertheless, another crucial aspect of processing point clouds in urban environments with PC is the possibility of filtering objects. This capability enables the individual accounting of buildings, cars, and vegetation, thereby facilitating more precise viewshed calculations. In contrast, DSM data does not offer such granularity, frequently resulting in overgeneralised visibility results.

The initial example demonstrated that the DSM omitted an architectural element, in this case a historic monument. Furthermore, the DSM failed to include any viewing windows that faced south from the point under analysis. This may have resulted in the city’s main viewing axis being overlooked. The second case demonstrated that in instances where the observer’s point is situated at a considerable height above the ground and surrounded by trees, the DSM chart encompasses a considerably wider range than is necessary. In the DSM, the value attributed to treetops is greater than in the PC. Following an analysis of the urban structures visible from the viaducts but located among the trees, the PC also identified structures that the DSM had overlooked due to generalization. The last cases demonstrate that the DSM is unable to assess viewing windows. In the case of selected point number 5, only the PC viewshed is able to present one of the city’s most significant monuments.

When comparing PC from LiDAR and DSM for graph generation, the Point Cloud allows for the creation of a detailed grayscale graph, highlighting the intensity of visibility areas with higher precision. In contrast, the DSM generates a bitmap graph where visibility is approximated. The PC graph offers more nuanced visibility information, while DSM is more generalized.

The article was derived from an example that demonstrated the importance of scale. In urban areas, it is crucial to determine visibility with higher accuracy. In the case of a larger area, where a pixel represents 2.56 m², both methods yield results that are quite similar. However, in an area where one pixel represents 0.36 m², the viewshed based on PC shows more detail, is more accurate, and omits generalization.

## Conclusion

The processing of point clouds is a far more computationally complex operation than that of data utilising a digital surface model, due to the significantly greater volume of data involved. As a result, they are unsuitable for use in large areas where the range of visibility can extend over long distances. Conversely, viewsheds based on this type of data yield results that are more accurate and include information that DSM may omit. Consequently, they are better suited to urban environments where the field of view is severely constrained by land cover features, particularly in the form of compact buildings.

It is important to note that the distinction between the urban landscape (townscape) and the open landscape was made as early as the 1960s [80]. Various methodologies for addressing these two categories of landscapes have been suggested, especially within the context of scenic analyses [81]. The validity of these approaches is confirmed by the research presented in this article. In the case of an open landscape, it is important that the viewshed covers a large area and helps to identify view connections on a macro scale, while the detail of the chart is slightly less important. In contrast, townscape is one area where accuracy in analysis is crucial.

Planning techniques have been employed for many centuries in a highly urbanised environment. The incorporation of scenic elements into these techniques serves to enhance the status of significant buildings on a city scale, thereby conferring the status of landmarks upon them. These include viewing axes employed since the Baroque era, which are directed at an object with an important urban function (landmark) or leave free space (foreground of the view), which allows the building to be observed from a proper perspective [82]. As a result, viewsheds created using PC are useful for research conducted at the city scale, whereas viewsheds generated using DSM are more appropriate in open spaces.

The distinction made in our conclusions is not solely based on computational demand but is grounded in a holistic assessment of the data quality, accuracy, and applicability for various scales and types of landscapes. For city-scale research, point clouds provide detailed and precise data, which is crucial for urban environments with complex structures. Conversely, DSMs are more appropriate for open spaces where the terrain’s general features are of greater interest and where DSMs can efficiently capture the broader landscape.

A multitude of disciplines capitalise on the versatility of methodologies, which are contingent on factors such as object size, computational complexity and the speed at which computation takes place. The domain of computer graphics provides an exemplar of this concept, wherein the idea of varying texture precision in accordance with the observer’s proximity is deployed (Level of Detail).

One of the main limitations affecting methods using point clouds is the number of points in the cloud, which can hinder data processing over larger areas. This limitation is particularly evident in high-density urban environments, where the point density can reach up to 12 points per square meter, compared to less dense suburban or rural areas where the density is around 6 points per square meter. Another significant limitation is the currency of the data. In high-density urban environments, data is frequently updated, which is crucial for accurate analysis. For example, in urban areas, the latest point cloud was created in 2023, whereas in less dense suburban or rural areas, the data is older, from 2019. The currency of the data has a significant impact on the accuracy of analyses and the quality of processed information, which is critical for decision-making and planning.

The next step will be to develop methods for working with non-simplified data based on LiDAR input for various applications. The goal is to accelerate the processing of this data and achieve a level where working with maximum accuracy is fast enough to make data processing widely accessible. This will allow the full potential of the data to be utilized, improving the quality of analyses and decision-making. Streamlining data processing will enable broader application across various fields, such as science, business, and public administration. Additionally, this research will contribute to developing a level-of-detail system for generating viewsheds, which will recommend where LiDAR or DSM should be used.
